# Integrated analysis of neuromuscular dysfunction and metabolic dysregulation in diabetic peripheral neuropathy: associations with digital deformities and clinical risk stratification in a case-control study

**DOI:** 10.3389/fphys.2026.1793602

**Published:** 2026-07-23

**Authors:** Esther Soler-Climent, Carmen Martinez-Corbalán, Erika Meléndez-Oliva, Jessica Román-Marroquí, Gema Prats-Boluda, Isabel Junquera-Godoy, Gemma González-Lorente, Jose Luis Martinez-de-Juan, Encarnación Agulló-García, Iciar Martin-Llaguno, Rosa María Cuadrado-Zaplana

**Affiliations:** 1 Health Department, Elche General Hospital, Elche, Alicante, Spain; 2 FISABIO, Valencia, Spain; 3 Department of Optics, Pharmacology and Anatomy, University of Alicante, Alicante, Spain; 4 Centro de Investigación e Innovación en Bioingeniería (Ci2B). Universitat Poltècnica de València., Valencia, Spain

**Keywords:** bioimpedance (BIA), diabetic peripheral neuropathy, digital deformities, hand dynamometry, surface electromyography, transfer entropy coefficient

## Abstract

**Background:**

Diabetic peripheral neuropathy (DPN) is a multifactorial complication in which metabolic dysregulation, altered body composition, and neuromuscular impairment may contribute to structural foot alterations and ulceration risk. These domains are often studied separately, limiting understanding of their combined relationship with digital deformities and diabetic-foot risk.

**Objective:**

To evaluate exploratory associations between digital deformities, diabetic-foot risk, and integrated anthropometric, metabolic, bioimpedance-derived, functional, and neuromuscular markers.

**Methods:**

This single-center case–control study included 65 adults: 28 with type 2 diabetes and 37 non-diabetic controls. Assessments included anthropometry, bioimpedance-derived parameters — phase angle (PhA), ECW/TBW ratio, and muscle quality — handgrip dynamometry, HbA1c, and the triglyceride–glucose (TyG) index. Diabetic-foot risk was classified according to IWGDF criteria in participants with type 2 diabetes. Analyses included Shapiro–Wilk tests, Student’s t-test or Mann–Whitney U test, Pearson or Spearman correlations, and one-way ANOVA with Tukey *post-hoc* tests. Missing data were below 5% and were imputed.

**Results:**

Diabetic-foot risk correlated with waist circumference (r = 0.443, p < 0.001), BMI (r = 0.333, p = 0.005), HbA1c (r = 0.560, p < 0.001), and TyG index (r = 0.454, p < 0.001). Digital deformities were associated with fasting glucose (r = 0.573, p < 0.001), HbA1c (r = 0.560, p < 0.001), and TyG index (r = 0.565, p < 0.001). Inverse associations were observed for handgrip strength (r = −0.340, p = 0.004), muscle quality (r = −0.311, p = 0.009), and PhA (r = −0.285, p = 0.018). Exploratory subgroup analyses showed differences across analytical risk strata for PhA (F = 6.99, p = 0.002), muscle quality (F = 6.99, p = 0.002), and handgrip strength (F = 4.04, p = 0.022).

**Conclusions:**

Digital deformities and diabetic-foot risk in DPN were associated with combined metabolic, bioimpedance-derived, and systemic neuromuscular alterations. Handgrip strength and bioimpedance-derived parameters showed exploratory associations with risk and deformities; however, their role as complementary assessment measures requires confirmation in larger longitudinal studies with adjusted models.

## Introduction

Diabetic peripheral neuropathy (DPN) is one of the most prevalent and disabling complications of diabetes mellitus, affecting up to 50% of patients during the course of the disease (Kimura et al., 2020; Chicharro-Luna et al., 2021b). It is characterized by progressive degeneration of peripheral nerve fibers involving sensory, motor, and autonomic components, leading to neuropathic pain, impaired proprioception, motor dysfunction, postural instability, and increased risk of diabetic-foot complications (Sarla, 2020; Chicharro-Luna et al., 2021a).

The clinical relevance of DPN lies in its strong association with foot ulceration, infection, and lower-limb amputation, which are frequently preceded or accompanied by structural foot alterations, including claw toes and hammer toes (Chicharro-Luna et al., 2021a; Favretto et al., 2022; Junquera-Godoy et al., 2024). These digital deformities modify plantar pressure distribution, increase focal mechanical stress, and contribute to tissue breakdown, thereby accelerating the pathway towards diabetic-foot complications (Moayedi et al., 2021; Junquera-Godoy et al., 2025).

From a pathophysiological perspective, DPN is a multifactorial disorder driven by chronic hyperglycemia, microvascular dysfunction, oxidative stress, mitochondrial impairment, advanced glycation end-product accumulation, and low-grade inflammation, which collectively induce neural damage and impair neuromuscular function (Singh et al., 2021). In addition to peripheral nerve injury, increasing evidence suggests the involvement of central nervous system alterations, including disrupted motor control and altered interoceptive processing, indicating that DPN may reflect a broader neurodegenerative process rather than an exclusively peripheral disorder (Aksu et al., 2024; Basebaa et al., 2024).

Neuromuscular dysfunction in DPN is characterized by progressive motor unit loss, denervation, impaired muscle contractility, and altered muscle activation patterns (Sawacha et al., 2012; Spolaor et al., 2016). These changes predominantly affect distal musculature, particularly the intrinsic foot muscles, which play a critical role in stabilizing foot architecture and maintaining digital alignment (Maranesi et al., 2016; Zang et al., 2023). Their deterioration may favor an imbalance between intrinsic and extrinsic musculature, resulting in unopposed forces that contribute to digital deformities such as claw toes and hammer toes (Parasoglou et al., 2017; Moayedi et al., 2021).

Structural and functional studies have demonstrated significant involvement of distal and intrinsic foot musculature in patients with DPN, including muscle atrophy, altered contractile function, and impaired neuromuscular control (Allen et al., 2014; Bodman et al., 2025). These changes may coexist with modifications in connective tissue structures, such as increased plantar aponeurosis thickness, further contributing to biomechanical dysfunction and altered load transmission within the foot (Parasoglou et al., 2017; Kimura et al., 2020).

From a biomechanical standpoint, digital deformities are associated with abnormal load redistribution and increased internal stresses within the foot during gait (Moayedi et al., 2021; Junquera-Godoy et al., 2025). Clinical and functional assessment approaches have highlighted the relevance of early identification of foot impairment in patients with diabetes, particularly before advanced structural complications become established (Bus et al., 2020; Manvitha et al., 2022).

Body composition and nutritional status may also contribute to the systemic phenotype of DPN. Bioimpedance-derived measures provide accessible information on body composition and tissue-related parameters, while sarcopenia-related frameworks emphasize the relevance of muscle strength and functional reserve in chronic disease populations (Cruz-Jentoft et al., 2019; Mundstock et al., 2021). Nutritional and immunometabolic status may further influence physical function and vulnerability, supporting the need to consider systemic markers beyond localized foot assessment (Di Vincenzo et al., 2023).

Sensorimotor impairment is another relevant dimension of DPN. Altered sensory-motor mechanisms increase fall risk and functional decline in individuals with DPN, suggesting that neuromuscular deterioration may extend beyond local foot structures (Reeves et al., 2021). In parallel, muscle morphology and electrophysiological alterations have been linked to neuropathic involvement, supporting the role of distal muscle impairment and axonal dysfunction in the progression of DPN-related functional limitations (Hansen and Ballantyne, 1977; Abdulkareem and Ameer, 2024).

Surface electromyography (sEMG) has emerged as a valuable tool for characterizing neuromuscular alterations in DPN. Beyond conventional amplitude- and frequency-based measures, advanced metrics such as transfer entropy (TE) and normalized mutual information (NMI) enable the assessment of nonlinear intermuscular connectivity, shared signal synchronization, and directional information flow between muscle groups (Junquera-Godoy et al., 2024; Junquera-Godoy et al., 2025). These approaches provide insight into neuromuscular network organization and its disruption in pathological conditions characterized by progressive motor system impairment.

Emerging evidence from previous analyses conducted in this cohort suggests that neuromuscular connectivity may follow a non-linear trajectory in DPN. In lower-risk or early stages, increased connectivity may reflect compensatory mechanisms and adaptive reorganization, whereas in more advanced stages, reduced connectivity may indicate progressive disintegration of motor control and impaired intermuscular coordination (Junquera-Godoy et al., 2024; Junquera-Godoy et al., 2025). This transition may contribute to the shift from functional adaptation to biomechanical instability and structural deterioration.

Despite these advances, most studies have investigated metabolic dysfunction, neuromuscular impairment, body composition, and biomechanical alterations as separate domains. However, DPN is unlikely to result from an isolated mechanism. Rather, metabolic dysregulation may contribute to neural injury and neuromuscular deterioration, while impaired muscle function may exacerbate biomechanical instability and favor the development of structural foot deformities (Sawacha et al., 2012; Parasoglou et al., 2017; Singh et al., 2021). This supports the need for integrative models capable of capturing the interaction between metabolic, cellular, functional, neuromuscular, and structural alterations.

**Figure 1:**
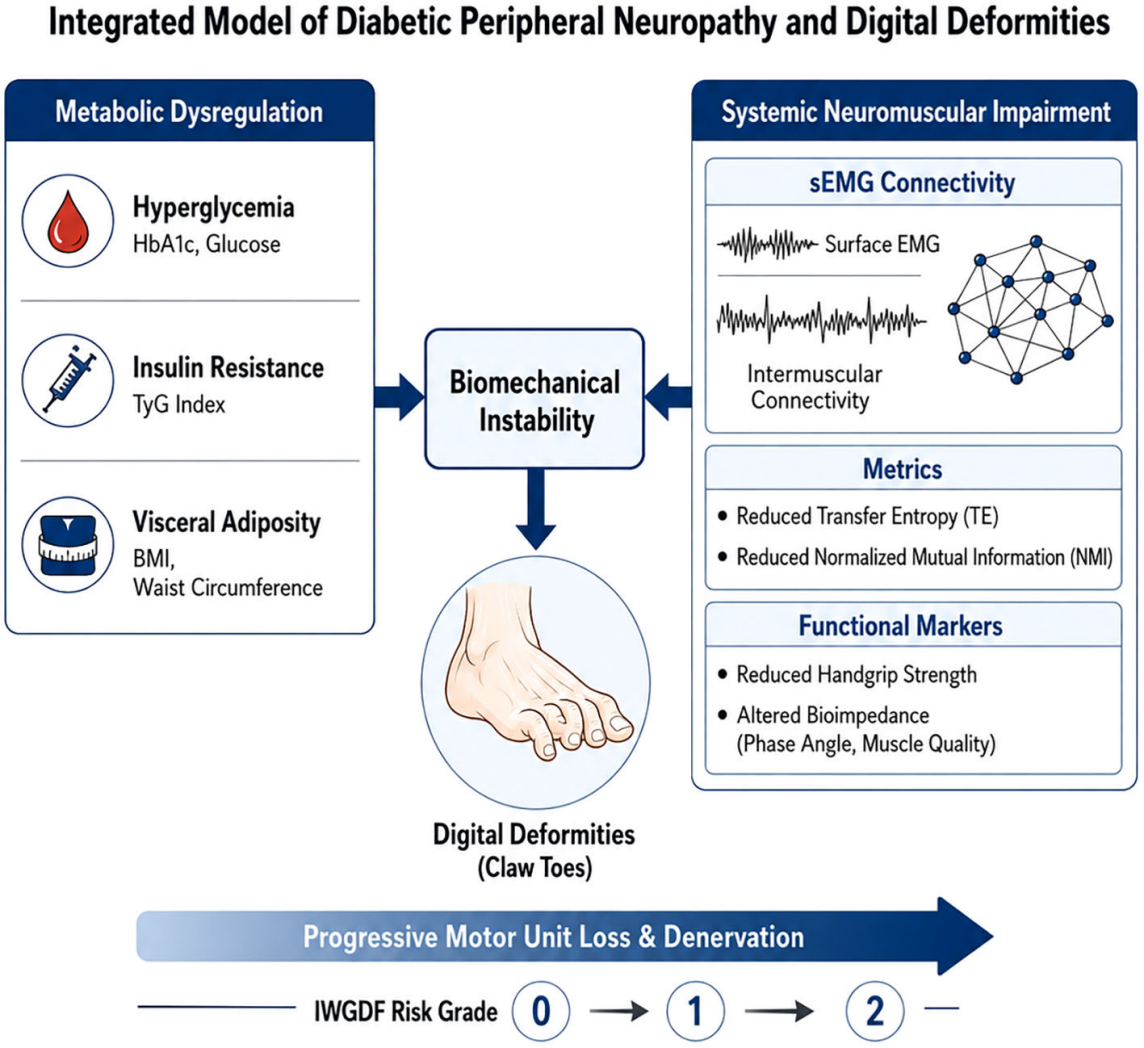
Integrated conceptual model linking metabolic dysregulation, neuromuscular impairment, and digital deformities in diabetic peripheral neuropathy. Metabolic alterations, including hyperglycemia, insulin resistance, visceral adiposity, and impaired cellular integrity, contribute to systemic neuromuscular dysfunction characterized by motor unit loss, denervation, altered sEMG patterns, reduced transfer entropy, reduced normalized mutual information (NMI), lower handgrip strength, and impaired bioimpedance-derived markers. These interconnected pathways converge toward biomechanical instability and act as a primary driver of systemic damage, promoting the development of digital deformities such as claw toes and increasing diabetic-foot risk across IWGDF categories.

Taken together, these findings support the need for an integrated analytical framework combining metabolic, bioimpedance-derived, functional, neuromuscular, and biomechanical domains to better understand the mechanisms underlying DPN-related digital deformities and diabetic-foot risk. The conceptual framework of the proposed model is illustrated in **Figure 1**, integrating metabolic dysregulation, altered cellular integrity, systemic neuromuscular impairment, biomechanical instability, and structural digital deformities within a unified exploratory framework.

Therefore, the present study aimed to evaluate exploratory associations between digital deformities, diabetic-foot risk, and an integrated set of anthropometric, metabolic, bioimpedance-derived, functional, and neuromuscular markers, including previously published intermuscular connectivity metrics derived from surface electromyography.

**Figure 1**. Conceptual exploratory framework integrating metabolic dysregulation, altered cellular integrity, systemic neuromuscular impairment, biomechanical instability, and digital deformities in diabetic peripheral neuropathy.

**Figure 1**.

## Method

### Design

This comparative case–control study was conducted at the General Hospital of Elche, Alicante, Spain, between March and June 2023. Participants were divided into two groups: individuals with type 2 diabetes, considered cases, and non-diabetic individuals, considered controls. The study aimed to explore neuromuscular, metabolic, and bioimpedance-derived markers associated with digital deformities and diabetic-foot risk.

Although the study focused on diabetic peripheral neuropathy (DPN)-related features and diabetic-foot risk, non-diabetic controls were included to provide a reference group for baseline metabolic, bioimpedance-derived, and neuromuscular parameters. This design may introduce confounding, because diabetes itself influences both metabolic and neuromuscular domains.

To partially address this limitation, analyses were complemented by stratification within the diabetic group according to the International Working Group on the Diabetic Foot (IWGDF) risk classification, grades 0–2 (Bus et al., 2020). This allowed exploratory comparisons across different stages of neuropathy-related risk within the same underlying population and enabled assessment of potential gradients associated with diabetic-foot risk severity. Future studies should include individuals with type 2 diabetes without neuropathy as a specific comparison group to better isolate DPN-specific mechanisms and improve causal interpretation.

Inclusion criteria for cases were diagnosis of type 2 diabetes for more than five years, age between 18 and 80 years, regular clinical follow-up, diabetic-foot risk grade 0, 1, or 2 according to the IWGDF classification (Bus et al., 2020), and availability of recent blood test results. Cases were also evaluated for the presence or absence of digital deformities, including claw toes and hammer toes, which were recorded as a distinct outcome.

Controls were non-diabetic individuals aged 18–80 years from the hospital’s healthcare coverage area, with available recent blood test results.

Exclusion criteria included difficulty walking or standing, prior use of plantar orthoses, presence of lower-extremity oedema, wounds at electrode placement sites, or diabetic-foot risk grade 3. Participants classified as IWGDF grade 3 were excluded because this category represents advanced diabetic-foot disease, defined by previous foot ulceration or lower-limb amputation.

Sample size was estimated using Epi Info™, based on the reference diabetic population of the study area and the expected prevalence of diabetic-foot complications. This ensured a minimum number of participants per group for exploratory comparative analyses. However, the study was not powered for confirmatory multivariable modelling, and findings should therefore be interpreted as exploratory.

### Data collection

Participants attended two visits within a three-day period. During the first visit, they received study information, provided written informed consent, underwent diabetic-foot assessment, and were assigned a risk grade according to IWGDF criteria (Bus et al., 2020).

IWGDF risk stratification was applied exclusively to participants with type 2 diabetes, as this classification is intended for persons with diabetes. In accordance with IWGDF criteria, grade 0 was defined as absence of loss of protective sensation and absence of peripheral artery disease; grade 1 as the presence of loss of protective sensation or peripheral artery disease; and grade 2 as loss of protective sensation plus peripheral artery disease and/or foot deformity.

The presence or absence of digital deformities was assessed by direct visual clinical inspection during the diabetic-foot examination. This assessment was performed by an experienced nurse with extensive clinical expertise in diabetic-foot risk evaluation and IWGDF-based risk classification. Digital deformities were recorded as present when clinically apparent structural alterations were observed, including claw toes, hammer toes, prominent metatarsal heads, or other visible forefoot deformities relevant to diabetic-foot risk and metatarsophalangeal joint dysfunction (Zellers et al., 2020). Deformities were coded dichotomously as present or absent; no quantitative severity grading, radiographic confirmation, or pressure-platform assessment was performed.

The second visit included body composition analysis through anthropometry and bioimpedance, handgrip dynamometry, retrieval of biochemical parameters from recent laboratory tests, and surface electromyography (sEMG) recordings of intrinsic foot muscles. Bioimpedance measurements were performed under standardized fasting and hydration conditions. Dynamometry followed a three-trial protocol using a calibrated device, with participants seated and the elbow flexed at 90°. sEMG acquisition followed standardized electrode placement and signal filtering procedures.

Surface electromyography-derived neuromuscular connectivity metrics were incorporated as part of an integrated analytical framework. Specifically, transfer entropy (TE), a nonlinear measure that quantifies the directionality and magnitude of information flow between time-series signals, and normalized mutual information (NMI), which evaluates the degree of shared information and synchronization between signals, were used to characterize intermuscular coordination.

These metrics are particularly relevant in the context of DPN, as they provide information on nonlinear intermuscular coordination, potential compensatory mechanisms, and progressive neuromuscular disorganization beyond conventional amplitude-based sEMG analyses.

Importantly, the sEMG-derived connectivity variables, including TE and NMI, were obtained from previously published analyses conducted on the same cohort (Junquera-Godoy et al., 2024; Junquera-Godoy et al., 2025) and were not newly computed for the present manuscript. No new sEMG signal processing or connectivity computation was performed for this study. Instead, these previously characterized variables were incorporated as neuromuscular biomarkers to enable their integration with newly analyzed metabolic, anthropometric, bioimpedance, handgrip, and digital deformity variables within a unified exploratory framework.

Therefore, the original contribution of the present study lies in the integration of previously published neuromuscular connectivity metrics with newly analyzed systemic and structural markers, rather than in the generation of new sEMG-derived variables.

The study protocol was approved by the Research Ethics Committee of the General Hospital of Elche (CEIm; protocol PI 138/2022), and all participants provided written informed consent in accordance with the Declaration of Helsinki.

### Variables

#### Outcome variables: diabetic-foot risk grade and digital deformities

The main outcome variables were diabetic-foot risk grade and the presence or absence of digital deformities. Diabetic-foot risk was classified according to IWGDF criteria in participants with type 2 diabetes (Bus et al., 2020). Digital deformities were recorded dichotomously as present or absent, as described above.

#### Bioimpedance-derived and body composition variables

Body composition was assessed using the TANITA MC-780MA Segmental Multi-Frequency Analyzer, which provided estimates of fat mass, fat-free mass, muscle mass, muscle quality, total body water, metabolic age, basal metabolic rate, and phase angle (PhA). Additional bioimpedance-derived metrics included the extracellular water/total body water ratio (ECW/TBW) and the extracellular mass/body cell mass ratio (ECM/BCM).

Phase angle was interpreted as an indirect marker of cellular integrity and membrane capacitance, reflecting the relationship between reactance and resistance. Lower phase angle values were considered indicative of poorer cellular health, reduced cell membrane integrity, and lower functional reserve. The ECW/TBW ratio was interpreted as an indicator of fluid distribution, with higher values suggesting relative extracellular water expansion, altered tissue hydration, or reduced tissue quality. Muscle quality was interpreted as a body-composition-derived estimate of muscle functional status, complementing muscle mass by providing information on the condition or efficiency of muscle tissue rather than muscle quantity alone.

Phase angle (PhA) was calculated using the following expression:

PhA = arctangent (reactance [Xc]/resistance [R]) × (180/π)

Together, these bioimpedance-derived parameters were used to provide an indirect assessment of cellular integrity, tissue hydration, and muscle functional status. In the context of DPN, lower PhA, higher ECW/TBW ratio, and reduced muscle quality were interpreted as potential indicators of impaired cellular health, altered fluid distribution, and systemic neuromuscular deterioration.

### Handgrip strength

Handgrip strength was measured as a proxy of global muscle function and systemic sarcopenia, given its established use as a functional marker of muscle strength and its relevance to generalized muscle weakness in diabetic neuropathy (Cruz-Jentoft et al., 2019; Van Eetvelde et al., 2021).

### Surface electromyography and neuromuscular connectivity

sEMG recordings of the intrinsic foot muscles were included to explore neuromuscular activation patterns that may relate to structural deformities, metatarsophalangeal joint instability, and compensatory gait strategies, given the role of intrinsic foot muscles in balance, turning, and forefoot structural control (Zellers et al., 2020; Taylor et al., 2022).

In addition to conventional sEMG parameters, intermuscular connectivity metrics such as TE and NMI were considered key variables reflecting network-level neuromuscular organization, enabling a more comprehensive characterization of motor control alterations in DPN.

### sEMG processing

sEMG was recorded using the Noraxon Ultium device. Signals were captured at 2000 Hz, applying a high-pass filter at 20 Hz and a low-pass filter at 500 Hz. The muscles monitored included the tibialis anterior, medial gastrocnemius, extensor digitorum brevis, and flexor digitorum brevis. The selection of intrinsic foot muscles for sEMG assessment was supported by previous evidence showing distal muscle involvement, intrinsic foot muscle atrophy, and altered lower-limb muscle morphology in DPN (Kishimoto et al., 2020; Sharath et al., 2024). The recorded signals were processed using MATLAB R2022b.

Intermuscular connectivity metrics, including TE and NMI, were considered as indicators of nonlinear coordination, shared information, and directional coupling between muscle pairs. These metrics were derived from previously processed sEMG signals and had been computed in prior analyses conducted on the same cohort, following validated methodologies in neuromuscular network analysis.

The methodological framework for sEMG acquisition, signal processing, and intermuscular connectivity analysis has been previously described in detail in studies conducted on this cohort (Junquera-Godoy et al., 2024; Junquera-Godoy et al., 2025). No new sEMG signal processing or connectivity computation was performed for the present manuscript. Instead, TE and NMI were incorporated as previously published neuromuscular biomarkers within an integrated analytical framework, allowing their combination with newly analyzed metabolic, bioimpedance, handgrip, and digital deformity variables.

### Statistical analysis

Sample size estimation was performed using Epi Info™. Data analysis was conducted using Jamovi software.

A structured analytical framework was applied, consisting of (1): descriptive analysis of clinical and demographic variables; (2) bivariate analyses using correlation and group comparison tests; and (3) exploratory subgroup analyses across diabetic-foot risk categories. This approach was designed to identify potential associations and gradients across metabolic, bioimpedance-derived, functional, and neuromuscular domains, rather than to establish independent predictors or confirm causal pathways.

Normality was assessed using the Shapiro–Wilk test. Continuous variables were summarized using means and standard deviations or medians and interquartile ranges, depending on their distribution. Categorical variables were summarized as frequencies and percentages. Group comparisons were performed using Student’s t-test or the Mann–Whitney U test, as appropriate. Correlation analyses were conducted using Pearson’s or Spearman’s coefficients according to data distribution. One-way ANOVA with Tukey *post-hoc* tests was applied for exploratory subgroup comparisons across risk categories.

Given the modest sample size and the number of variables assessed, no confirmatory multivariable regression models were specified. Although adjustment for potential confounders such as age, sex, BMI, body composition, and diabetes duration would be desirable, extensive adjusted modelling in this dataset would have increased the risk of overfitting and unstable estimates. Therefore, the results should be interpreted as exploratory and hypothesis-generating. Future studies should incorporate larger samples with predefined adjusted models to determine whether the observed associations are independent of relevant clinical and demographic covariates.

### Handling missing data

Missing data were below 5% across the dataset. The distribution of missing values was inspected by variable and study group, and no systematic concentration of missingness was observed in either participants with type 2 diabetes or non-diabetic controls. Missingness was considered compatible with a missing completely at random (MCAR) pattern.

Missing values were handled using Multiple Imputation by Chained Equations (MICE). The imputation model included demographic, anthropometric, metabolic, bioimpedance-derived, handgrip, diabetic-foot risk, and digital deformity variables to preserve the multivariable structure of the dataset.

Given the small proportion of missing data, imputation was not expected to materially alter the direction of the main associations. However, formal sensitivity analyses comparing imputed and complete-case datasets were not performed; this has been acknowledged as a limitation.

## Results

### Sample characteristics

In the final sample of 65 participants, 28 (43.1%) had type 2 diabetes and 37 (56.9%) were included in the control group. Overall, 38.6% of participants were male and 61.4% were female. Regarding age distribution, 42.0% were between 45 and 64 years old, 39.1% were older than 65 years, and 18.8% were aged 44 years or younger.

With respect to educational background, 7.2% of participants had no formal education, 40.6% had completed primary education, 26.1% had completed secondary education, and 26.1% had attained a university degree. Regarding income level, 79.7% reported a medium income, while 20.3% reported a low income.

Among the 28 participants with type 2 diabetes, all underwent complete diabetic-foot risk assessment according to the International Working Group on the Diabetic Foot (IWGDF) criteria (Bus et al., 2020). The distribution was as follows: 10 participants (35.7%) were classified as low risk, 6 (21.4%) as moderate risk, and 12 (42.9%) as high risk. None of the participants in the low-risk group showed clinically apparent signs of diabetic peripheral neuropathy (DPN), whereas all individuals in the moderate/high-risk group exhibited clinical signs compatible with DPN.

### Biomarker analysis

All analyses were conducted in the full sample (N = 65), including both participants with type 2 diabetes and non-diabetic controls. For exploratory analytical purposes, non-diabetic controls were included within the lowest analytical reference category because they had no diabetes, no evidence of peripheral neuropathy, no peripheral arterial disease, and no clinically apparent structural foot alterations. This approach was used to explore gradients across metabolic, bioimpedance-derived, functional, and neuromuscular domains, but it should not be interpreted as assigning a true clinical IWGDF grade 0 status to non-diabetic individuals, since the IWGDF diabetic-foot risk classification is intended for persons with diabetes.

Anthropometric and metabolic parameters showed statistically significant associations with diabetic-foot risk. Waist circumference showed a moderate positive correlation (r = 0.443, p < 0.001), as did body mass index (BMI) (r = 0.333, p = 0.005) and visceral fat index (r = 0.293, p = 0.015). Glycemic control markers were also associated with risk, particularly glycated hemoglobin (HbA1c) (r = 0.560, p < 0.001) and fasting glucose (r = 0.532, p < 0.001). The triglyceride–glucose (TyG) index was significantly correlated with risk (r = 0.454, p < 0.001), consistent with its use as a surrogate marker of insulin resistance (**Table 1**).

**Table 1:** Correlations between the studied variables and the Diabetic Foot Risk for the whole sample (N=65).

Predictive variable	Correlation	P value^a^	R^2^	P value^b^
Relaxed arm circumference	0.064	0.604	0.0040	0.604
Waist circumference	0.443	<0.001**	0.1960	<0.001**
Non-Dominant Hand DYN	-0.340	0.004**	0.0786	0.019*
Glucose	0.532	<0.001**	0.2620	<0.001**
HbA1c	0.560	<0.001**	0.3400	<0.001**
Triglycerides	0.345	0.004**	0.0674	0.034*
TyG index	0.454	<0.001**	0.1910	<0.001**
BMI	0.333	0.005**	0.0968	0.009**
Visceral Fat Index	0.293	0.015*	0.0756	0.022*
Sarcopenia Risk Index	0.178	0.143	0.0159	0.302
CONUT	0.430	0.008**	0.2810	<0.001**
PA	-0.332	0.005**	0.0969	0.009**
Basal Metabolism (Kcal)	0.068	0.580	0.0045	0.581
Metabolic Age	0.370	0.002**	0.1370	0.002**
Age	0.299	0.012*	0.0833	0.015*
Muscular Quality	-0.311	0.009**	0.0969	0.009**
Tot. Body Water (%)	-0.264	0.028*	0.0697	0.028*

DPN, diabetic peripheral neuropathy; IWGDF, International Working Group on the Diabetic Foot; PA, phase angle; ECW/TBW, extracellular water/total body water; ECM/BCM, extracellular mass/body cell mass; TyG, triglyceride–glucose index; CONUT, Controlling Nutritional Status score; sEMG, surface electromyography; TE, transfer entropy; NMI, normalized mutual information. Correlation analyses were performed considering the full sample; inclusion of non-diabetic controls within risk grade 0 may enhance variability in the low-risk range.

^a^ P values by Pearson’s coefficient for normal variables and Spearman’s for non-normal variables.

^b^ P values from linear regression.Bold values indicate statistically significant associations or group differences. * p < 0.05; ** p < 0.01.

**Table 1**.

Handgrip strength, used as a proxy of global neuromuscular function, showed statistically significant associations with several body composition variables. Positive correlations were observed with lean mass (r = 0.395, p = 0.001), fat-free mass (r = 0.381, p = 0.001), intracellular water (r = 0.542, p < 0.001), and phase angle (PhA) (r = 0.331, p = 0.003). Conversely, the extracellular-to-total body water ratio (ECW/TBW) was inversely correlated with handgrip strength (r = −0.548, p < 0.001), suggesting that altered fluid distribution may be associated with reduced muscle function (**Table 2**).

**Table 2:** Correlations between the studied variables and Non-Dominant Hand Dynamometry (DYN) for the whole sample (N=65).

Predictive variable	Correlation	P value^a^	R^2^	P value^b^
Risk Grade of Diabetic Foot	-0.340	0.004**	0.078	0.019*
Age	-0.344	0.004**	0.091	0.011*
Arm Circumference	0.065	0.594	0.033	0.138
Calf Circumference	0.23	0.058*	0.101	0.008*
4-Meter Walking Speed	-0.338	0.005**	0.093	0.013*
HbA1c	-0.21	0.097	0.031	0.166
BMI	-0.002	0.989	0.024	0.207
Fat-Free Mass	0.381	0.001**	0.126	0.003**
Lean Mass	0.395	0.001**	0.293	<0.001**
Muscle Quality	0.395	<0.001**	0.126	0.003**
Sarcopenia Risk Index	0.381	<0.001**	0.251	<0.001**
Total Body Water	0.471	<0.001**	0.23	<0.001**
Extracellular Water	0.333	0.005**	0.098	0.009*
Intracellular Water	0.542	<0.001**	0.37	<0.001**
ECW/TBW Ratio	-0.548	<0.001**	0.296	<0.001**
ECM/BCM Ratio	-0.351	0.003**	0.083	0.017*
Bone Mineral Content	0.455	<0.001**	0.287	<0.001**
Visceral Fat Index	0.126	0.301	0.094	0.011*
Basal Metabolic Rate	0.447	<0.001**	0.284	<0.001**
PA	0.331	0.003**	0.109	0.003**

BMI, body mass index; ECW/TBW ratio, extracellular water/total body water ratio; ECM/BCM ratio, extracellular mass/body cell mass ratio; HbA1c, glycated hemoglobin; PA, phase angle. Correlation analyses were performed considering the full sample; inclusion of non-diabetic controls within risk grade 0 may enhance variability in the low-risk range.

^a^ P values by Pearson’s coefficient for normal variables and Spearman’s for non-normal variables.

^b^ P values from linear regression.Bold values indicate statistically significant associations or group differences. * p < 0.05; ** p < 0.01.

**Table 2**.

Phase angle analysis showed statistically significant associations with metabolic and functional variables. Negative correlations were observed with age (r = −0.547, p < 0.001), metabolic age (r = −0.477, p < 0.001), HbA1c (r = −0.251, p = 0.047), and CONUT score (r = −0.457, p = 0.005), while positive associations were found with handgrip strength (r = 0.331, p = 0.003) and muscle quality. These findings are consistent with the interpretation of PhA as an indirect marker related to cellular integrity, metabolic status, and functional reserve (**Table 3**).

**Table 3:** Correlations between the studied variables and PA for the whole sample (N=65).

Predictive variable	Correlation	P value^a^	R2	P value^b^
Relaxed arm circumference	0.336	0.005**	0.113	0.005**
Non-Dominant Hand DYN	0.331	0.003**	0.109	0.003**
HbA1c	-0.251	0.047*	0.0682	0.039*
Triglycerides	0.075	0.547	0.0009	0.811
TyG index	0.054	0.666	0.0006	0.837
BMI	0.091	0.457	0.2333	0.211
Visceral Fat Index	0.049	0.688	0.0004	0.871
Sarcopenia Risk Index	0.330	0.006**	0.0926	0.011*
CONUT	-0.457	0.005**	0.244	0.002**
Basal Metabolism (Kcal)	0.350	0.003**	0.102	0.007**
Metabolic Age	-0.477	<0.001**	0.160	<0.001**
Age	-0.547	<0.001**	0.258	<0.001**
Muscular Quality	1.000	<0.001**	0.999	<0.001**
Tot. Body Water (%)	0.391	<0.001**	0.123	0.003**

BMI, body mass index; CONUT, controlling nutritional status; DYN, dynamometry; HbA1c, glycated hemoglobin; TyG index: triglyceride glucose index. Correlation analyses were performed considering the full sample; inclusion of non-diabetic controls within risk grade 0 may enhance variability in the low-risk range.

^a^ P values by Pearson’s coefficient for normal variables and Spearman’s for non-normal variables.

^b^ P values from linear regression.Bold values indicate statistically significant associations or group differences. * p < 0.05; ** p < 0.01.

**Table 3**.

From a nutritional and inflammatory perspective, the Controlling Nutritional Status (CONUT) score showed a positive association with diabetic-foot risk (r = 0.430, p = 0.008), suggesting a possible relationship between impaired immunonutritional status and higher risk categories.

Neuromuscular and bioimpedance-derived parameters were inversely associated with diabetic-foot risk. Handgrip strength was negatively correlated with risk (r = −0.340, p = 0.004), as were muscle quality (r = −0.311, p = 0.009) and PhA (r = −0.332, p = 0.005). These findings suggest that reduced muscular performance and altered bioimpedance-derived parameters may be associated with increasing diabetic-foot risk severity.

Digital deformities, including claw toes and prominent metatarsal heads, showed statistically significant associations with metabolic dysregulation. Fasting glucose (r = 0.573, p < 0.001), HbA1c (r = 0.560, p < 0.001), triglycerides (r = 0.434, p < 0.001), and TyG index (r = 0.565, p < 0.001) were significantly correlated with deformity presence. Body composition parameters such as BMI (r = 0.239, p = 0.048) and visceral fat index (r = 0.304, p = 0.011) were positively associated, while PhA (r = −0.285, p = 0.018) and muscle quality (r = −0.311, p = 0.009) showed inverse associations. These findings are consistent with a potential relationship between systemic metabolic and bioimpedance-derived alterations and structural foot changes (**Table 4**).

**Table 4:** Correlations between the studied variables and the claw toes or prominent metatarsal heads for the whole sample (N=65).

Predictive variable	Correlation	P value^a^	R^2^	P value^b^
Relaxed arm circumference	0.336	0.005**	0.113	0.005**
Non-Dominant Hand DYN	-0.34	0.004**	0.0786	0.019*
Glucose	0.573	<0.001**	0.295	<0.001**
HbA1c	0.56	<0.001**	0.34	<0.001**
Triglycerides	0.434	<0.001**	0.137	0.002*
TyG index	0.565	<0.001**	0.314	<0.001**
BMI	**0.239**	0.048*	0.084	0.016*
Visceral Fat Index	0.304	0.011*	0.019	0.254
Sarcopenia Risk Index	0.187	0.124	0.065	0.035*
CONUT	0.345	0.037*	0.19	0.007*
PA	-0.285	0.018*	0.075	0.023*
Basal Metabolism (Kcal)	0.35	0.003**	0.102	0.007*
Metabolic Age	-0.477	<0.001**	0.16	<0.001**
Age	-0.547	<0.001**	0.258	<0.001**
Muscular Quality	-0.311	0.009*	0.0969	0.009*
Tot. Body Water (%)	0.391	<0.001**	0.123	0.003**

BMI, body mass index; CONUT, controlling nutritional status; DYN, dynamometry; HbA1c, glycated hemoglobin; PA, phase angle; TyG index: triglyceride glucose index. Correlation analyses were performed considering the full sample; inclusion of non-diabetic controls within risk grade 0 may enhance variability in the low-risk range.

^a^ P values by Pearson’s coefficient for normal variables and Spearman’s for non-normal variables.

^b^ P values from linear regression.Bold values indicate statistically significant associations or group differences. * p < 0.05; ** p < 0.01.

**Table 4**.

Group comparisons using one-way ANOVA across analytical reference categories suggested a possible gradient of deterioration. Significant differences were observed for PhA (F = 6.99, p = 0.002), muscle quality (F = 6.99, p = 0.002), and handgrip strength (F = 4.04, p = 0.022), with *post-hoc* analyses showing lower values in higher-risk groups. These findings should be interpreted as exploratory, given the case–control design, modest sample size, and absence of multivariable adjustment (**Table 5**).

**Table 5:** One-Way ANOVA (Fisher’s) and Tukey Post-Hoc Test.

Variables	ANOVA		*Post Hoc*	
	F	P value^a^	Comparisons	P value^b^
PA	6.99	0.002 **	C vs 0	0.999
			C vs 1-2	0.002 **
			0 vs 1-2	0.029 *
Fat Mass (%)	3.42	0.039 *	C vs 0	0.985
			C vs 1-2	0.036 *
			0 vs 1-2	0.210
Fat-Free Mass (%)	3.24	0.045 *	C vs 0	0.996
			C vs 1-2	0.044 *
			0 vs 1-2	0.205
Muscle Quality	6.99	0.002 **	C vs 0	0.999
			C vs 1-2	0.002 **
			0 vs 1-2	0.029 *
Total Body Water (%)	3.79	0.028 *	C vs 0	0.996
			C vs 1-2	0.027 *
			0 vs 1-2	0.156
Metabolic Age	5.53	0.006 **	C vs 0	0.956
			C vs 1-2	0.008 **
			0 vs 1-2	0.037 *
Non-Dominant Hand DYN	4.04	0.022*	C vs 0	0.758
			C vs 1-2	0.0089 **
			0 vs 1-2	0.031 *

DPN, diabetic peripheral neuropathy; IWGDF, International Working Group on the Diabetic Foot; PA, phase angle; ECW/TBW, extracellular water/total body water; ECM/BCM, extracellular mass/body cell mass; TyG, triglyceride–glucose index; CONUT, Controlling Nutritional Status score; sEMG, surface electromyography; TE, transfer entropy; NMI, normalized mutual information.

Risk grade 0 includes both non-diabetic controls and diabetic participants without neuropathy, as neither group presented neuropathy, vasculopathy, or structural foot alterations. This approach was used to increase the reference low-risk group for exploratory analyses. Results should therefore be interpreted within the context of this classification strategy.

^a^ P value ANOVA Test.

^b^ P value Tukey post-hoc test.Bold values indicate statistically significant associations or group differences. * p < 0.05; ** p < 0.01.

**Table 5**.

### Neuromuscular connectivity analysis

Surface electromyography (sEMG)-derived neuromuscular connectivity metrics were included to characterize patterns of intermuscular coordination associated with diabetic-foot risk. Specifically, transfer entropy (TE), a nonlinear measure of directional information flow, and normalized mutual information (NMI), an index of shared signal synchronization, were considered as previously published sEMG-derived neuromuscular connectivity metrics obtained from analyses conducted on the same cohort (Junquera-Godoy et al., 2024; Junquera-Godoy et al., 2025).

In line with previous analyses conducted in this cohort (Junquera-Godoy et al., 2024; Junquera-Godoy et al., 2025), a progressive alteration in neuromuscular connectivity was observed across risk groups. Individuals classified as lower risk according to the diabetic-foot analytical risk categories exhibited higher TE and NMI values, suggesting increased intermuscular coordination and potential compensatory mechanisms. In contrast, participants in higher-risk categories demonstrated reduced connectivity, consistent with impaired motor unit synchronization and progressive neuromuscular disorganization.

These findings are consistent with previously published results from the same cohort (Junquera-Godoy et al., 2024; Junquera-Godoy et al., 2025), where alterations in sEMG amplitude and frequency patterns were interpreted as reflecting motor unit loss and denervation processes. In the present study, sEMG-derived connectivity metrics were not reanalyzed but incorporated as previously published neuromuscular biomarkers within an integrated analytical framework combining metabolic, anthropometric, bioimpedance-derived, handgrip, and digital deformity variables.

## Discussion

This discussion builds on previously published findings from the same cohort (Junquera-Godoy et al., 2024; Junquera-Godoy et al., 2025), in which surface electromyography (sEMG) analysis demonstrated a reduction in signal amplitude in the tibialis anterior and extensor digitorum brevis muscles, accompanied by an increase in mean frequency, consistent with progressive denervation and motor unit loss. Additionally, significant alterations in intermuscular connectivity were reported, with transfer entropy (TE) showing high discriminative capacity for detecting diabetic peripheral neuropathy (DPN), even at early stages. In particular, TE between the medial gastrocnemius and intrinsic foot muscles differentiated risk groups, revealing increased connectivity in lower-risk individuals and reduced connectivity in higher-risk groups, reflecting progressive neuromuscular disorganization.

Transfer entropy (TE), a nonlinear metric that quantifies the direction and magnitude of information flow between physiological signals, and normalized mutual information (NMI), which reflects the degree of shared information and synchronization between muscle activations, provide complementary insights into neuromuscular network organization (Junquera-Godoy et al., 2024; Junquera-Godoy et al., 2025). These metrics extend beyond conventional amplitude-based sEMG analyses by capturing coordination and connectivity patterns, which are particularly relevant in conditions such as DPN, characterized by progressive motor system disintegration and broader central nervous system involvement, including altered intrinsic brain activity related to neurological and motor dysfunction (Xin et al., 2022; Zang et al., 2023).

In the present study, these sEMG-derived connectivity findings were not reanalyzed but incorporated as previously published neuromuscular biomarkers obtained from the same cohort (Junquera-Godoy et al., 2024; Junquera-Godoy et al., 2025), allowing their integration with newly analyzed metabolic, anthropometric, bioimpedance, handgrip, and digital deformity variables within a broader systemic framework. This approach avoids methodological redundancy and enables the contextualization of previously characterized neuromuscular network alterations alongside systemic and structural markers.

Accordingly, the novelty of the present manuscript does not lie in the generation of new sEMG connectivity metrics, but in the integration of previously published neuromuscular connectivity markers with newly analyzed metabolic, anthropometric, bioimpedance, handgrip, and digital deformity variables. This distinction has been clarified to avoid ambiguity regarding which analyses were newly conducted and which were incorporated from prior work.

Building on these observations, the present study expands the analytical framework by examining the relationship between metabolic dysregulation, altered bioimpedance-derived parameters, systemic neuromuscular impairment, and digital deformities. Although the cross-sectional design precludes causal inference, the convergence of metabolic, bioimpedance, handgrip, digital deformity, and neuromuscular connectivity findings is consistent with a biologically plausible pathway in which chronic hyperglycemia and insulin resistance may contribute to peripheral nerve injury, impaired motor unit recruitment, intrinsic foot muscle dysfunction, and progressive imbalance between intrinsic and extrinsic musculature.

In this framework, digital deformities such as claw toes should not be interpreted solely as local mechanical consequences of repetitive microtrauma or abnormal loading. Rather, they may reflect the structural expression of a broader neuromuscular process in which denervation, reduced muscle quality, impaired intermuscular coordination, and altered tissue integrity interact with biomechanical stress. Repetitive loading, reduced protective sensation, and abnormal plantar pressure may still contribute to structural progression, but the observed pattern of associations supports the plausibility of an integrated pathophysiological interpretation involving metabolic, neuromuscular, and biomechanical domains.

### Mechanistic interpretation of the integrated model

From a mechanistic perspective, chronic hyperglycemia and insulin resistance may promote neural injury through oxidative stress, microvascular dysfunction, advanced glycation end-product accumulation, mitochondrial impairment, and low-grade inflammation (Zhu et al., 2024). These processes can affect both sensory and motor fibers, contributing to impaired proprioception, reduced protective sensation, altered motor unit behavior, and progressive denervation. Motor involvement is particularly relevant for digital deformities because intrinsic foot muscles are essential for maintaining toe alignment and metatarsophalangeal joint stability.

As intrinsic muscle function deteriorates, extrinsic flexor and extensor forces may become relatively dominant, favoring progressive digital imbalance. This imbalance may contribute to claw toe or hammer toe deformities, which in turn increase focal plantar pressures and biomechanical stress. In parallel, lower handgrip strength, reduced phase angle, altered ECW/TBW ratio, and lower muscle quality may indicate that DPN-related impairment is not restricted to the foot, but may reflect a systemic neuromuscular phenotype. Therefore, the integrated pattern observed in this study is biologically coherent with a model in which metabolic dysregulation, cellular impairment, neuromuscular dysfunction, and biomechanical instability interact in the development of DPN-related structural foot alterations.

This interpretation is aligned with the conceptual framework shown in **Figure 1**, which integrates metabolic dysregulation, altered cellular integrity, neuromuscular impairment, biomechanical instability, and digital deformities within a unified explanatory model. However, **Figure 1** should be interpreted as an exploratory integrative framework derived from the convergence of the present findings and previous evidence, rather than as a confirmed causal model. Its purpose is to visually organize the proposed relationships between metabolic, bioimpedance-derived, functional, neuromuscular, and structural domains, while acknowledging that longitudinal validation is required to determine the temporal sequence and causal relevance of these pathways.

These results are consistent with previous studies demonstrating that DPN is associated with altered muscle activation patterns and impaired motor coordination. Abnormalities in gait mechanics and muscle activation timing have been described in individuals with diabetes, supporting the presence of early neuromuscular dysfunction (Sawacha et al., 2009; Sawacha et al., 2012). Similarly, altered electromyographic patterns during functional tasks have been observed in both neuropathic and non-neuropathic diabetic populations, suggesting that motor impairment may extend beyond clinically overt neuropathy (Akashi et al., 2008; Spolaor et al., 2016). From a biomechanical perspective, finite element analyses have shown that deformities such as claw toes lead to abnormal internal stress redistribution within the foot, reinforcing the link between structural deformity and mechanical overload (Moayedi et al., 2021).

Importantly, previous research has typically evaluated metabolic dysfunction, neuromuscular impairment, and biomechanical alterations as independent domains. The present findings align with emerging evidence suggesting that integrated models may better explain the progression of DPN and its structural consequences, as neuromuscular deterioration and metabolic dysregulation are mechanistically interconnected rather than independent processes (Zhu et al., 2024; Soler-Climent et al., 2025).

Our findings are consistent with the concept that digital deformities may not be merely a consequence of localized muscle wasting, but may also reflect a broader systemic neuromuscular imbalance. The combination of intrinsic muscle impairment, reduced global strength, altered bioimpedance parameters, and disrupted intermuscular connectivity supports the plausibility of a loss of coordinated motor control, in which relative extrinsic muscle dominance may contribute to progressive biomechanical instability and structural deformity (Bohnert et al., 2020; Kimura et al., 2020; Zellers et al., 2020).

Intrinsic muscle atrophy appears to be a central factor in the development of digital deformities. Imaging, ultrasound, and distal muscle studies have demonstrated progressive alterations in intrinsic and distal foot muscles, including reduced muscle thickness, altered morphology, and extensor digitorum brevis atrophy, contributing to altered foot structure and biomechanics (Kishimoto et al., 2020; Abdulkareem and Ameer, 2024; Sharath et al., 2024).

In line with these findings, our results showed associations between digital deformities and markers of metabolic dysregulation, including HbA1c, blood glucose, and the triglyceride–glucose (TyG) index. This pattern is consistent with the concept that chronic hyperglycemia and insulin resistance may contribute to neuromuscular degeneration and structural deterioration through mechanisms involving oxidative stress, microvascular dysfunction, mitochondrial impairment, and low-grade inflammation (Zhu et al., 2024). However, given the cross-sectional design, these findings should be interpreted as mechanistically plausible associations rather than evidence of a confirmed causal sequence.

From a systemic perspective, the observed inverse association between handgrip strength and diabetic-foot risk suggests that neuromuscular dysfunction in DPN may not be limited to the lower extremities, but may reflect a more generalized pattern of muscle weakness involving both distal and proximal muscle groups (Van Eetvelde et al., 2021). Similarly, reductions in phase angle and muscle quality may indicate impaired cellular integrity, altered tissue composition, and reduced functional reserve, reinforcing the concept of whole-body involvement and supporting the relevance of bioimpedance-derived phase angle as an indicator of diabetic polyneuropathy in type 2 diabetes (Cruz-Jentoft et al., 2019; Mundstock et al., 2021; Schimpfle et al., 2024).

The group comparisons further suggested a possible gradient of progressive neuromuscular impairment across International Working Group on the Diabetic Foot (IWGDF) risk categories, with significant reductions in strength and bioimpedance-derived parameters in higher-risk groups. These findings are consistent with the hypothesis that functional decline may begin before advanced structural complications become evident and highlight the importance of early detection of subtle functional and neuromuscular alterations in DPN (Salmen et al., 2020; Reeves et al., 2021).

From a methodological perspective, the integration of previously published sEMG connectivity metrics with newly analyzed metabolic, anthropometric, bioimpedance, handgrip, and digital deformity variables represents a key strength of this study. This combined approach allows the identification of systemic patterns that may not be detectable when analyzing each domain independently, thereby providing a more comprehensive interpretation of the mechanisms potentially involved in DPN-related structural foot alterations.

The observed associations should nevertheless be interpreted with caution because of the modest sample size and the absence of multivariable adjustment. Potential confounders such as age, sex, BMI, body composition, and diabetes duration may have influenced the relationships identified between metabolic dysregulation, bioimpedance-derived parameters, handgrip strength, digital deformities, and diabetic-foot risk. Therefore, these findings should not be considered evidence of independent associations, but rather exploratory signals that support the need for larger, adequately powered studies with predefined adjusted models.

From a clinical standpoint, the integrated model proposed in this study provides added value over traditional approaches by combining metabolic, neuromuscular, and body composition markers into a unified exploratory framework. This approach may help generate hypotheses regarding the early systemic features associated with digital deformities and functional deterioration, although its clinical applicability requires confirmation in longitudinal studies.

Clinically, this integrative framework may support a shift from localized foot assessment toward a broader evaluation of neuromuscular and metabolic health. In particular, accessible tools such as handgrip dynamometry and bioimpedance may warrant further investigation as complementary assessment measures, but they should not be considered validated screening or monitoring instruments without prospective validation.

These findings may also inform future intervention strategies. A combined approach including optimized glycemic control, targeted muscle strengthening, and neuromotor training could be biologically plausible for addressing the multifactorial mechanisms involved in DPN-related functional decline. Interventions such as resistance exercise and sensorimotor training have shown potential benefits in improving muscle function and coordination in individuals with DPN (Holmes and Hastings, 2021; Rajan, 2024). However, further research is needed to determine optimal protocols, long-term efficacy, and whether such interventions can modify the trajectory of digital deformities or diabetic-foot risk.

Overall, this study supports the plausibility of an integrated pathophysiological model in which digital deformities in DPN arise from the interaction between metabolic dysregulation, systemic neuromuscular impairment, altered cellular integrity, and biomechanical instability. Although causal relationships cannot be established from the present cross-sectional design, the convergence of findings across metabolic, bioimpedance, functional, structural, and neuromuscular domains provides a coherent framework for future longitudinal research.

### Study limitations and future research

This study has several limitations that should be considered when interpreting the findings. First, the case–control design and single-center setting may limit generalizability and introduce selection bias. In addition, the modest sample size, although adequate for exploratory analyses, limits statistical power and restricts the feasibility of more complex multivariable modeling. Therefore, the findings should be interpreted as hypothesis-generating rather than confirmatory.

Second, the inclusion of non-diabetic controls requires careful interpretation. In the present study, non-diabetic participants were included as the lowest analytical reference category to provide a baseline comparator for metabolic, bioimpedance-derived, and neuromuscular parameters. However, these individuals should not be interpreted as a true clinical IWGDF grade 0 group, because the IWGDF diabetic-foot risk classification is specifically intended for persons with diabetes. Although this reference-based approach allowed exploratory assessment of progressive differences across the full study sample, diabetes itself may influence metabolic and neuromuscular status. Consequently, residual confounding cannot be excluded. Future studies should include a specific group of individuals with type 2 diabetes without neuropathy to better isolate DPN-specific mechanisms and improve internal validity.

Third, potential confounders such as age, sex, BMI, body composition, and diabetes duration were not controlled through multivariable regression models. Given the limited sample size and the number of variables assessed, extensive adjusted modelling would have increased the risk of overfitting and unstable estimates. Nevertheless, the absence of multivariable adjustment limits the ability to determine whether the observed associations are independent of these covariates.

Fourth, digital deformities were assessed by direct visual clinical inspection and recorded as present or absent. This assessment was performed by an experienced nurse with expertise in diabetic-foot risk evaluation and IWGDF-based risk classification. However, no radiographic confirmation, pressure-platform assessment, or quantitative severity grading was performed. This may reduce reproducibility and comparability with studies using objective or graded measures of claw toes, hammer toes, or metatarsophalangeal deformities.

Fifth, neuromuscular assessment relied on sEMG-derived connectivity metrics and handgrip dynamometry. Although these tools provide relevant information on neuromuscular coordination and systemic muscle function, they may not fully capture the complexity of lower-limb motor dysfunction in DPN. Moreover, the sEMG-derived transfer entropy and normalized mutual information metrics were obtained from previously published analyses conducted on the same cohort and incorporated here as neuromuscular biomarkers within an integrated analytical framework. Therefore, the present study should not be interpreted as an independent replication or new computation of these sEMG-derived variables. Rather, its contribution lies in integrating these previously characterized neuromuscular metrics with newly analyzed metabolic, bioimpedance-derived, handgrip, and digital deformity variables.

Sixth, bioimpedance-derived parameters, including phase angle, ECW/TBW ratio, and muscle quality, may be influenced by hydration status, nutritional state, inflammation, medication use, and other physiological factors. Although measurements were performed under standardized conditions, these variables should be interpreted as indirect markers of cellular integrity, tissue hydration, and muscle functional status rather than as disease-specific biomarkers.

Seventh, missing data were below 5% and were handled using multiple imputation. However, the small sample size limits the robustness of imputation procedures, and the possibility of unrecognized missing-data patterns cannot be fully excluded. Formal sensitivity analyses comparing imputed and complete-case datasets were not performed; this should be addressed in future studies with larger datasets.

Finally, the cross-sectional nature of the study precludes causal inference and does not allow determination of whether metabolic dysregulation, altered bioimpedance-derived parameters, reduced muscle strength, or neuromuscular connectivity changes precede the development of digital deformities or increased diabetic-foot risk. Longitudinal studies with larger samples, diabetic non-neuropathic comparison groups, standardized deformity grading, and predefined adjusted statistical models are needed to evaluate temporal relationships and to determine whether markers such as handgrip strength, phase angle, ECW/TBW ratio, muscle quality, or sEMG-derived connectivity metrics have predictive or clinical utility in DPN risk stratification.

## Conclusion

This study suggests that diabetic peripheral neuropathy is associated with systemic alterations involving metabolic dysregulation, impaired cellular integrity, reduced muscle strength, and altered neuromuscular connectivity. Digital deformities and diabetic-foot risk showed exploratory associations with glycemic imbalance, TyG index, reduced phase angle, altered ECW/TBW ratio, lower muscle quality, and handgrip strength.

Although the cross-sectional design precludes causal inference, the convergence of metabolic, bioimpedance-derived, functional, structural, and neuromuscular findings supports the plausibility of an integrated pathophysiological framework linking chronic metabolic dysregulation, systemic neuromuscular impairment, biomechanical instability, and digital deformities.

Handgrip strength, phase angle, ECW/TBW ratio, muscle quality, and sEMG-derived connectivity metrics should not be interpreted as validated predictive biomarkers, diagnostic tools, or screening markers. Their potential role as complementary assessment measures requires confirmation in larger longitudinal studies including diabetic non-neuropathic comparison groups, standardized deformity grading, and predefined adjusted statistical models.

## Data Availability

The raw data supporting the conclusions of this article will be made available by the authors, without undue reservation.
